# Multi-parameter immune profiling of peripheral blood mononuclear cells by multiplexed single-cell mass cytometry in patients with early multiple sclerosis

**DOI:** 10.1038/s41598-019-55852-x

**Published:** 2019-12-19

**Authors:** Chotima Böttcher, Camila Fernández-Zapata, Stephan Schlickeiser, Desiree Kunkel, Axel R. Schulz, Henrik E. Mei, Carl Weidinger, René M. Gieß, Susanna Asseyer, Britta Siegmund, Friedemann Paul, Klemens Ruprecht, Josef Priller

**Affiliations:** 1 Charité – Universitätsmedizin Berlin, corporate member of Freie Universität Berlin, Humboldt-Universität zu Berlin, and Berlin Institute of Health, Berlin, Germany; 2 Department of Neuropsychiatry and Laboratory of Molecular Psychiatry, Berlin, Germany; 3 BIH Center for Regenerative Therapies (BCRT), Berlin, Germany; 4 German Rheumatism Research Center (DRFZ), Berlin, Germany; 5 Division of Gastroenterology, Infectiology and Rheumatology, Medical Department for Gastroenterology, Berlin, Germany; 6 Department of Neurology and Clinical and Experimental Multiple Sclerosis Research Center, Berlin, Germany; 7 NeuroCure Clinical Research Center (NCRC), Berlin, Germany; 8 Berlin Institute of Health, Berlin, 10178 Germany; 9 Experimental and Clinical Research Center (ECRC), Max Delbrueck Center for Molecular Medicine, Berlin, Germany; 10 DZNE, Berlin Germany, University of Edinburgh and UK Dementia Research Institute, Edinburgh, UK

**Keywords:** Neuroimmunology, Multiple sclerosis

## Abstract

Multiple sclerosis (MS) is an inflammatory demyelinating and neurodegenerative disease of the central nervous system (CNS). Studies in rodent models demonstrated an association of CNS-infiltrating monocyte-derived macrophages with disease severity. However, little is known about humans. Here, we performed an exploratory analysis of peripheral blood mononuclear cells (PBMCs) isolated from healthy controls and drug-naïve patients with early MS using multiplexed single-cell mass cytometry and algorithm-based data analysis. Two antibody panels comprising a total of 64 antibodies were designed to comprehensively analyse diverse immune cell populations, with particular emphasis on monocytes. PBMC composition and marker expression were overall similar between the groups. However, an increased abundance of CCR7^+^ and IL-6^+^ T cells was detected in early MS-PBMCs, whereas NFAT1^hi^T-bet^hi^CD4^+^ T cells were decreased. Similarly, we detected changes in the subset composition of the CCR7^+^ and MIPβ^hi^ HLA-DR^+^ lymphocyte compartment. Only mild alterations were detected in monocytes/myeloid cells of patients with early MS, namely a decreased abundance of CD141^hi^IRF8^hi^CXCR3^+^CD68^−^ dendritic cells. Unlike in Crohn’s disease, no significant differences were found in the monocyte fraction of patients with early MS compared to healthy controls. This study provides a valuable resource for future studies designed to characterise and target diverse PBMC subsets in MS.

## Introduction

Multiple sclerosis (MS) is a frequent chronic inflammatory demyelinating and neurodegenerative disease of the human central nervous system (CNS) with a highly variable disease course^1^. Although its precise aetiology remains to be identified, peripheral immune cells have been proposed as one of the main players in MS. Indeed, immunotherapies targeting lymphocytes or subsets thereof (for example natalizumab, rituximab/ocrelizumab, alemtuzumab or fingolimod) have a beneficial effect on relapse rates and neuroimaging outcomes in patients with relapsing-remitting MS, while the effects of these drugs during the progressive phase of the disease are less pronounced^2–4^. Besides the adaptive immune system, the innate arm including monocytes has also been claimed to be involved in MS pathogenesis and/or disease severity. In mouse models of inflammatory demyelination (namely experimental autoimmune encephalomyelitis, EAE), CCR2^+^Ly6-C^hi^ blood monocytes were shown to exert a crucial role in the effector phase of the disease. Inhibition of their recruitment to the CNS strongly reduced EAE severity^5,6^. In humans, genome-wide association studies (GWAS) unravelled causal variants for MS in multiple cell types including different subsets of T and B cells, monocytes and macrophages, suggesting contributions of both adaptive and innate arms of the immune system^7^. Yet, the roles of monocytes/myeloid cells as well and their phenotypic changes in human MS remain elusive.

In humans, peripheral blood monocytes can be classified into three main subsets: classical (CD14^++^CD16^−^), intermediate (CD14^+^CD16^+^) and nonclassical (CD14^dim/−^CD16^+^) monocytes^8^. Results obtained from whole-genome expression arrays revealed that both CD14^+^ subsets (namely classical and intermediate monocytes) resemble CCR2^+^Ly6C^hi^ “inflammatory” monocytes in mice, whereas the nonclassical subset was more alike to mouse CX3CR1^hi^Ly6C^lo^ “patrolling” monocytes^8^. However, functional characteristics of human CD14^++^CD16^−^ and CD14^+^CD16^+^ monocytes revealed significant differences^8,9^. CD14^++^CD16^−^ classical monocytes strongly produce reactive oxygen species (ROS), IL-6, IL-8, CCL2 and CCL3, whereas the CD14^+^CD16^+^ intermediate subset predominantly produces TNF-α and IL-1β^8^. Phenotypic analysis by flow cytometry identified elevated expression of CD40, CD86 and CCR2 in nonclassical and intermediate monocytes from MS patients, while only slight differences were observed in the classical CD14^+^ subset^10,11^. However, another flow cytometry study found a reduction of CD40, CD163 and CCR2 expression in monocytes from MS patients^12^. These controversies may result from different strategies of the subset identification based on up to 8 markers, as well as the experimental *in vitro* conditions. In particular, the limited number of markers applied for immune profiling using flow cytometry renders it virtually impossible to simultaneously investigate the MS-associated responses of monocytes in comparison to other immune cell subsets such as T and B cells, which are known key players in MS. Massive immune cell profiling using multiplexed single-cell mass cytometry (CyTOF) allows for comprehensive investigation of various immune cell subsets. Commonly, up to 40 markers can be simultaneously investigated at the single-cell level, and this provides an important advantage over the classical flow cytometric analysis. Furthermore, the identification of immune cell subsets using an unbiased algorithm-based approach allows for the investigation of rare cell populations, which may otherwise remain unidentified on the basis of a hierarchical two-dimensional gating strategy.

In this study, we employed multiplexed CyTOF and algorithm-based data processing and analysis for high-dimensional immune cell profiling of PBMCs in early MS, with a particular emphasis on monocytes. We herein report the results of simultaneous analysis of monocyte/myeloid subsets and other immune cell populations in PBMCs (excluding granulocytes) from drug-naïve patients with early MS in comparison to healthy controls. Our findings provide a valuable resource for immune cell identification and profiling in future preclinical and clinical studies in early MS.

## Results

The demographic and clinical data of the patients with early MS and healthy controls included in this study are summarized in Supplementary Table 1. Gender and age did not differ between patients with early MS and healthy controls [*sex (% female)*: CON = 90.9, early MS = 72.7, *p* = 0.5865; *age (years* ± *SD)*: CON = 36 ± 12, early MS = 35 ± 9, t_(df = 20)_ = 0.0599, *p* = 0.9529].

### Comprehensive phenotyping by multiplexed single-cell mass cytometry

**Figure 1:**
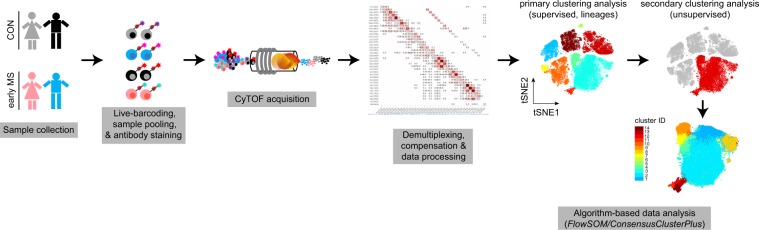
Schematic representation of CyTOF measurement. Peripheral blood mononuclear cells (PBMCs) were collected from healthy controls (CON, n = 11) and patients with early multiple sclerosis (MS) (early MS, n = 11). PBMCs were CD45-barcoded and pooled. Mixed samples were equally divided and stained with two panels (*Panel A* and *B*, Supplementary Tables 2 and 3) of metal-conjugated antibodies and acquired on the CyTOF instrument. Prior to algorithm-based data analysis, the data were demultiplexed and compensated. Two steps of clustering analysis were performs; the primary supervised clustering analysis to identify the known lineage cell subsets, and the secondary unsupervised clustering analysis to discover small phenotypic differences within each identified lineage cell subset.

The experimental design of this study is summarized in Fig. 1. In order to minimize the run-to-run variation and to facilitate the comparison of cellular profiles from different cell subsets and individuals, we simultaneously profiled PBMCs of all samples from both early MS patients and healthy controls (CON) in the same run. Isolated PBMCs from patients with early MS and healthy controls were thawed and live (mass-tag) barcoded using pre-conjugated CD45 antibody conjugates^13,14^. Samples were subsequently pooled, split equally and stained with two different antibody panels (35 antibodies/panel) (Supplementary Tables 2 and 3). *Panel A* was designed to detect the major circulating immune cell subsets (i.e. T & B cells, monocytes, natural killer (NK) cells), chemokine receptors and inflammatory mediators, including IRF4, IRF8, CD45, CD3, CD14, CD16, CD62L, CD19, HLA-DR, CD56, CD44, CD33 (Siglec-3), NFAT1, ADRP, CCR2, CCR7, IL-10, CCL2, IFN-γ, and TNF-α. *Panel B* was designed to investigate functional and activity changes in immune cell subsets using 35 antibodies including CD116, IKZF1, CD38, MIPβ, CD172a, PD-L1, Arginase-1, GATA6, GM-CSF, IRF8, GLUT1, IL-4, IL-8. In both antibody panels, anti-HLA-DR, anti-CD8a and anti-CD33 antibodies were included, which allowed tracking and correlation of immune phenotypes (revealed from both panels) of the myeloid cell populations between panels. Finally, multiplexed and stained samples were simultaneously acquired on a CyTOF instrument.

**Figure 2:**
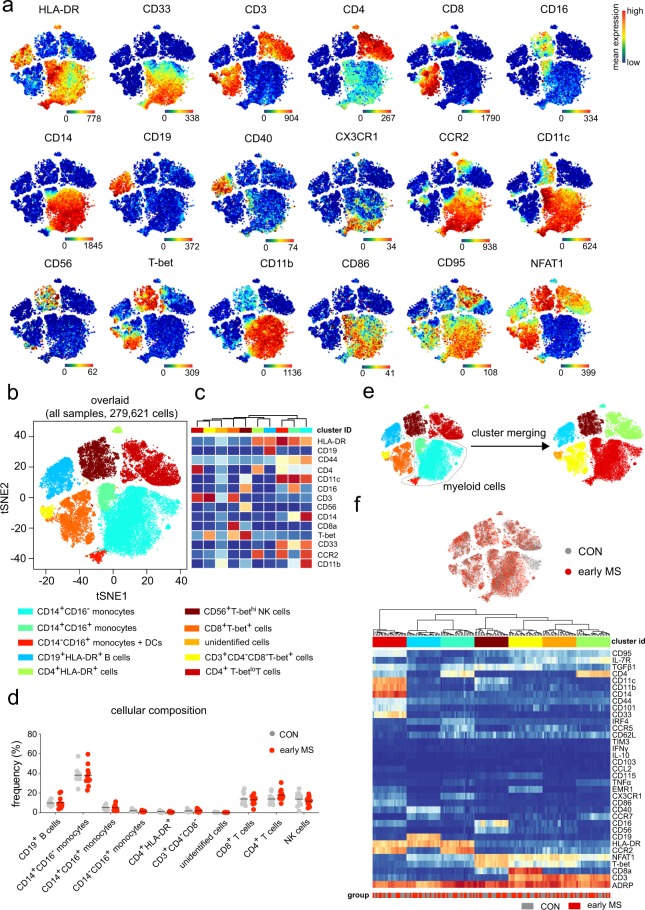
Immune phenotyping of peripheral blood mononuclear cells (PBMCs) – *Panel A*. (**a**) Representative reduced-dimensional single-cell t-SNE maps (of a healthy control) from biologically independent samples of patients with early MS (n = 11) and healthy controls (n = 11). Each dot represents one cell. The 2D t-SNE maps were generated based on expression levels of all markers of *Panel A* (Supplementary Table 2). The colour spectrum represents individual marker-expression levels (red, high expression; dark blue, no expression). (**b**) The t-SNE plot of concatenated FCS files from all 22 samples. The colouring indicates ten defined clusters representing major PBMC-lineages. (**c**) Heat map cluster demonstrates the expression levels of 14 markers used for the cluster analysis. (**d**) Quantified frequencies (%) of each defined cell subset showing comparable cellular composition in PBMCs from the two studied groups (black lines show mean values of the datasets). (**e**) Myeloid clusters including CD14^+^CD16^−^, CD14^+^CD16^+^, CD14^−^CD16^+^ monocytes and dendritic cells were manually merged prior to further data analysis. (**f**) Overlaid t-SNE plot shows cellular distribution of control (grey dots) and early MS (red dots) samples (top image). Heat map and cluster analysis of all samples on the basis of the mean expression of 36 markers. Samples are indicated by dendrograms. Heat colours show overall expression levels (red, high expression; dark blue, no expression).

**Figure 3:**
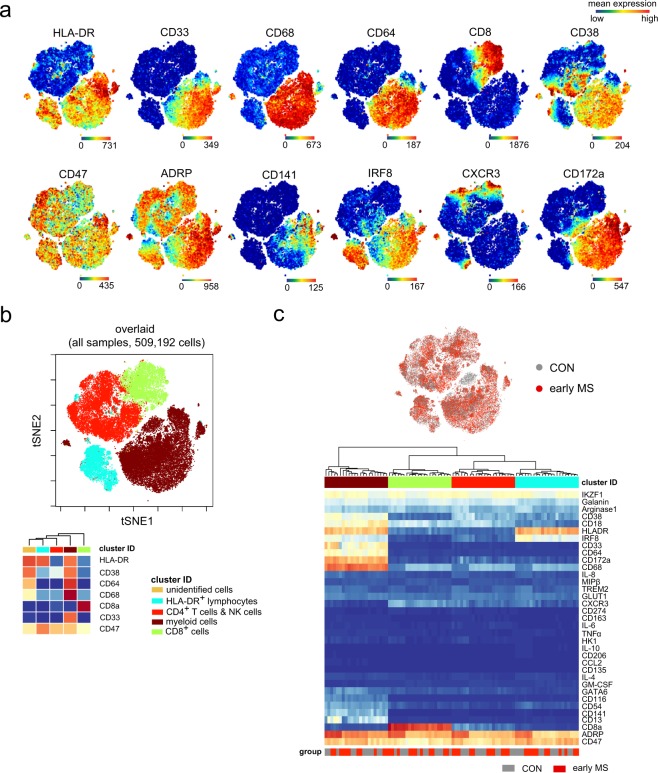
Immune phenotyping of peripheral blood mononuclear cells – *Panel B*. (**a**) Representative single-cell t-SNE plots (of a healthy control). Each dot represents one cell. The 2D t-SNE maps were generated based on expression levels of all markers of *Panel B* (Supplementary Table 3). The colour spectrum represents expression levels (red, high expression; dark blue, no expression). (**b**) The t-SNE map of concatenated FCS files from all 22 samples. The colouring indicates five defined clusters of myeloid and lymphoid origin. The lower panel shows cluster heat map cluster demonstrating the expression levels of 7 markers used as the embedding parameters. (**c**) Overlaid t-SNE plot shows cellular distribution of control (grey dots) and early MS (red dots) samples (top image). Heat map and cluster analysis of all samples on the basis of the mean expression of 36 markers. Samples are indicated by dendrograms. Heat colours show overall marker expression levels (red, high expression; dark blue, no expression).

First, we captured and visualized all cell subpopulations in a single two-dimensional (2D) map using unsupervised high-dimensional data analysis, the t-distributed stochastic linear embedding (t-SNE) algorithm^15,16^ on the commercially available analysis platform Cytobank (www.cytobank.org) (Figs. 2a and 3a). Subsequently, we performed a comprehensive analysis on R/Bioconductor by high resolution testing for differential abundance at the t-SNE embedding^17^ as well as by clustering-based methodologies^18^. Using antibody *Panel A*, in which HLA-DR, CD19, CD44, CD4, CD11c, CD16, CD3, CD56, CD14, CD8a, T-bet, CD33, CCR2 and CD11b were used for meta-clustering, we detected nine distinct clusters and a dispersed cluster containing only very few cells (less than 50 cells), which showed unclear phenotypic profiles (named as unidentified cells). These cells were excluded from the further data analysis (Fig. 2b,c). The immune cell composition of PBMCs was overall similar between the patients with early MS and healthy controls (Fig. 2d). To obtain enough resolution for further analysis, we manually merged some clusters, which resulted in a final of six clusters representing B cells, CD4/CD8-single positive and double-negative T cells, NK cells, and myeloid cells (Fig. 2e). These subsets served as input for further meta-clustering analysis. Using antibody *Panel B*, in which only HLA-DR, CD38, CD64, CD68, CD8a, CD33 and CD64 were used to estimate the frequency of the main circulating cell subsets, four cell subsets were defined. Using this antibody panel, we aimed to particularly analyse the myeloid cell population (HLA-DR^+^CD33^+^). Myeloid cells can be clustered from other cell populations (Fig. 3b), whereas CD4^+^ T cells could not be clearly clustered from NK cells, and some HLA-DR^+^ T lymphocytes (red dots) were detected in the cluster of B cells (HLA-DR^+^CD33^−^, light blue cluster). Similar to *Panel A*, one very rare cell subset (unidentified cells) was excluded from the analysis (Fig. 3b). In both *Panels A and B*, overall mean expressions of all markers used in *Panels A and B* were not different between the two groups (Figs. 2f and 3c).

### Phenotypic alterations in the lymphoid compartment in patients with early MS

**Figure 4:**
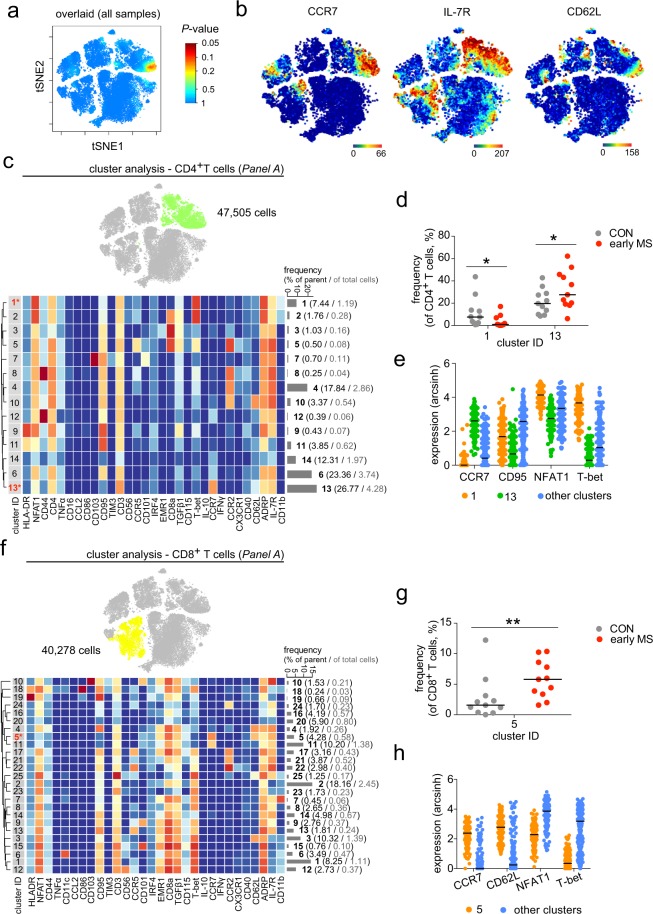
Phenotypic analysis of the lymphoid lineage. (**a**) Statistical t-SNE map obtained from bin-wise testing for differential abundance between the two studied groups (CON, n = 11; early MS, n = 11) using *edgeR* statistical framework (with negative binomial GLM) and 10% FDR adjustment. The colour spectrum corresponds to FDR-adjusted P values, ranging from 1 (blue) to ≤ 0.05 (red), detecting significant differences between the group in the CD4^+^ T cell subset. (**b**) Representative single-cell t-SNE map indicates the expression of CCR7, IL-7R and CD62L. The colour spectrum represents an expression level (red, high expression; dark blue, no expression). (**c**) t-SNE map highlights CD4^+^ T cell subset (green dots). Heat map and cluster analysis within the CD4^+^ T cell cluster from all samples on the basis of the mean expression of analysed markers. Identified clusters are indicated by dendrograms. Heat colours show overall marker expression levels. The bar graph shows mean cluster frequencies as % of parent (black number) and % of total cells (grey number). Cluster 1 and 13 (* in red) are quantified as differentially abundant subsets between the groups. (**d**) The graph shows differences in frequency (%) of cluster 1 and 13 between the two studied groups. (**e**) Median expression levels as shown in dot plot representation for randomly selected cells (n = 256/cluster) in the cluster 1 (orange), 13 (green) and all other clusters (blue). The selected markers are identified as defining markers for cluster 1 and 13. (**f**) The t-SNE map, heat map and cluster analysis of CD8^+^ T cells from all samples on the basis of the mean expression of analysed markers. Cluster 5 (* in red) is quantified as differentially abundant subsets between the groups. (**g**) The graph shows differences in frequency (%) of cluster 5 between the two studied groups. (**h**) Median expression levels as shown in dot plot representation for randomly selected cells (n = 256/cluster) in the cluster 5 (orange) and all other clusters (blue). A *P* value < 0.05 at 10% FDR was considered statistically significant, determined using GLMM (*P < 0.05; **P < 0.01, unadjusted).

**Figure 5:**
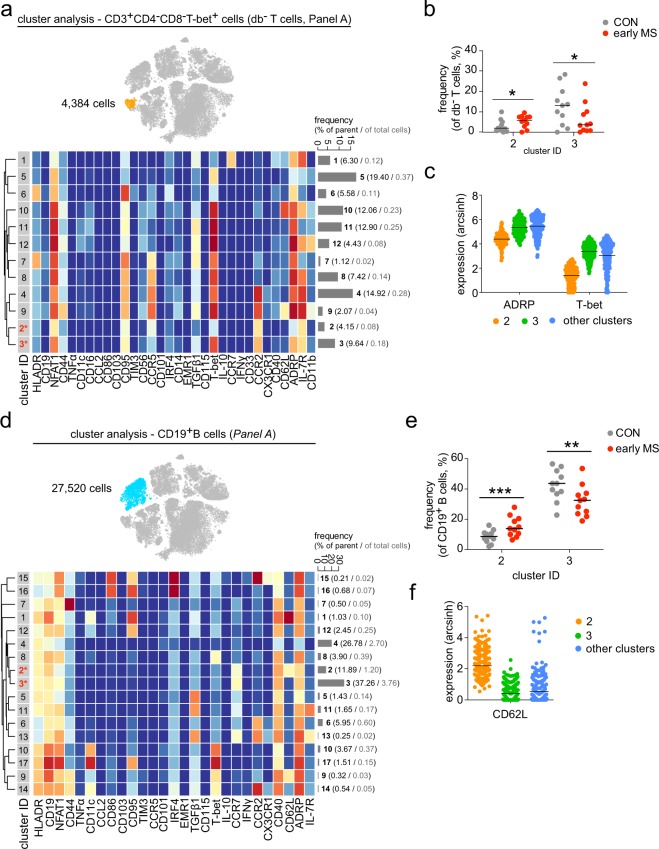
Cluster analysis of CD3^+^CD4^−^CD8^−^Tbet^+^ T cells and B cells from early MS patients (early MS) compared to the healthy controls (CON). (**a**) t-SNE map highlights CD3^+^CD4^−^CD8^−^Tbet^+^ T cell subset (orange dots). Heat map and cluster analysis of CD3^+^CD4^−^CD8^−^Tbet^+^ T cells from all samples on the basis of the mean expression of analysed markers. Identified clusters are indicated by dendrograms. Heat colours show overall marker expression levels (red, high expression; dark blue, no expression). The bar graph shows mean cluster frequencies as % of parent (black number) and % of total cells (grey number). Cluster 2 and 3 (* in red) are quantified as differentially abundant subsets between the healthy controls and the early MS patients. (**b**) The graph shows differences in frequency (%) of the three subsets between the two studied groups (CON, grey; early MS, red). (**c**) Median expression levels as shown in dot plot representation for randomly selected cells (n = 256/cluster) in the cluster 2 (orange), 3 (green), and all other clusters (blue). ADRP and T-bet are identified as defining markers for cluster 2 and 3. (**d**) t-SNE map highlights CD19^+^ B cell subset (blue dots). Heat map and cluster analysis of CD19^+^ B cells from all samples on the basis of the mean expression of analysed markers. Identified clusters are indicated by dendrograms. Heat colours show overall marker expression levels (red, high expression; dark blue, no expression). Cluster 2 and 3 (* in red) are quantified as differentially abundant subsets between healthy controls and patients with early MS. (**e**) The graph shows differences in frequency (%) of cluster 2 and 3 between the two studied groups (CON, grey; early MS, red). (**f**) Median expression levels as shown in dot plot representation for randomly selected cells (n = 256/cluster) in the cluster 2 (orange), 3 (green) and all other clusters (blue). CD62L was statistically detected as a main marker defining the differentially abundant cluster 2 and 3. A *P* value < 0.05 at 10% FDR was considered statistically significant, determined using GLMM (*P < 0.05; **P < 0.01; ***P < 0.001, unadjusted).

In order to examine phenotypic differences of PBMCs between patients with early MS and healthy controls, we next performed high-resolution probability binning on the t-SNE map of the entire data set, an approach that has been shown to be an effective exploring strategy to reveal differences between the studied groups^17^. This analysis revealed that the CD4^+^ T cell composition was different between healthy controls and patients with early MS (Fig. 4a). The differences were detected in the CD4^+^ T cell sub-populations that expressed CCR7, IL-7R and CD62L (Fig. 4b). Further cluster analysis of CD4^+^ T cells indicated abundant differences in two out of fourteen subsets (Fig. 4c,d). Cluster 1, defined as NFAT1^hi^T-bet^hi^CD4^+^ T cells was present at a lower frequency in patients with early MS compared to healthy controls (Fig. 4d,e), whereas the abundance of CCR7^+^T-bet^−^CD4^+^ T cells (cluster 13) was higher in patients with early MS (Fig. 4d,e**)**. Similar to the CD4^+^ lymphocytes, an increased frequency of the CCR7^+^T-bet^−^NFAT1^lo^ cell subset (cluster 5) was also found among the CD8^+^ T cell population (Fig. 4f–h). Within the CD3^+^CD4^−^CD8^−^T-bet^+^ (double-negative) T cells, we detected differential abundance in two subsets 2 and 3, which differed from the other clusters on the basis of expression levels of ADRP, CCR2 and T-bet (Fig. 5a–c). Notably, in the early MS group, increased cell frequencies were detected in the clusters that were ADRP^lo^CCR2^+^T-bet^lo^ (cluster 2), while ADRP^+^CCR2^+^T-bet^+^ cells (cluster 3) were less abundant compared with healthy controls (Fig. 5a–c).

Although we could not detect differential marker expression and abundance in the total B cell population, high-dimensional clustering analysis revealed differences in cell frequencies of two out of seventeen B cell subsets (Fig. 5d–f). These subsets were identified as CCR7^+^CCR2^−^TGFβ1^−^ B cells, which were positive (cluster 2) and negative (cluster 3) for CD62L (L-selectin) expression (Fig. 5d–f). Interestingly, we detected an increased abundance of CD62L-expressing B cells, whereas the frequency of the CD62L-negative cells was reduced (Fig. 5e,f).

**Figure 6:**
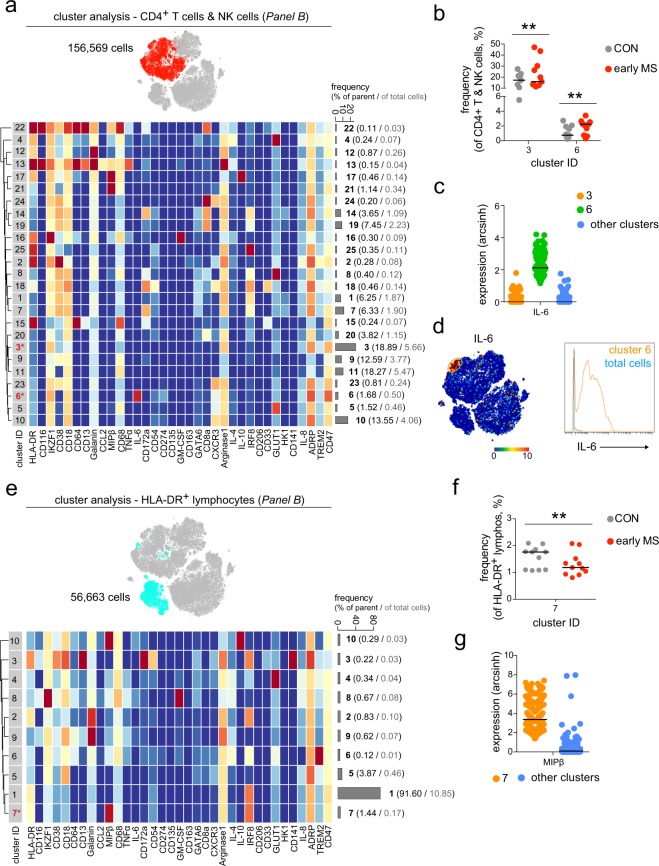
Differential abundances of lymphoid cell subsets in patients with early MS. (**a**) t-SNE map highlights mixed T and NK cell population (red dots). Heat map and cluster analysis of the mixed CD4^+^ T cell and NK cell population from all samples on the basis of the mean marker expressions (*Panel B*). Clusters are indicated by dendrograms. Heat colours show marker expression levels (red, high expression; dark blue, no expression). The bar graph shows mean cluster frequencies as % of parent (black number) and % of total cells (grey number). Cluster 3 and 6 (* in red) show differential abundances between healthy controls and patients with early MS. (**b**) The graph shows differences in frequency (%) of cluster 3 and 6 between the two studied groups (CON, grey; early MS, red). (**c**) Median expression levels as shown in the dot plot representation for randomly selected cells (n = 256/cluster) in the cluster 3 (orange), 6 (green) and all other clusters (blue). IL-6 was defined as a marker discriminating the differentially abundant cluster 6. (**d**) The representative t-SNE plot shows IL-6 expression levels and indicates the IL-6-expressing cell subset (orange circle, left image). The right image shows expression levels of IL-6 expressing cluster 6 (orange line), compared to the total cells (blue line). (**e**) t-SNE map highlights HLA-DR^+^ lymphocyte population (light blue dots). Heat map and cluster analysis of B cells from all samples on the basis of the mean marker expressions (*Panel B*). Cluster 7 (* in red) show differential abundances between the studied groups. (**f**) The graph shows differences in frequency (%) of cluster 7 between the two studied groups (CON, grey; early MS, red). (**g**) Median expression levels as shown in the dot plot representation for randomly selected cells (n = 256/cluster) in the cluster 7 (orange) and all other clusters (blue). MIPβ was identified as a marker discriminating the differentially abundant cluster 7. A *P* value < 0.05 at 10% FDR was considered statistically significant, determined using GLMM (**P < 0.01, unadjusted).

Similar to the results obtained for antibody *Panel A*, multi-parameter immune profiling using the antibody *Panel B* revealed differential abundances of lymphoid cell subsets (Fig. 6). In the mixed cluster of CD4^+^ T cells and NK cells, we detected differences in the frequencies of two cell subsets, namely clusters 3 and 6 in Fig. 6a,b. A unique CD47^hi^IL-6^+^ T cell subset (cluster 6) was found to be increased in early MS patients compared to the healthy controls (Fig. 6a–c). This subset could be further defined as CD274^lo^CD54^lo^CXCR3^+^ (Fig. 6a). Finally, the frequency of cells expressing high level of MIPβ (CCL4) and IRF8 (cluster 7) was significantly lower in the mixed cluster of HLA-DR^+^ T cells and B cells from patients with early MS than in healthy controls (Fig. 6d–f). In sum, harnessing the full power of multi-dimensional mass cytometry we unravelled phenotypic alterations of lymphoid cell subsets in patients with early MS compared to healthy controls. The changes in immune profiles detected herein were found mainly in the B and T cell populations.

### Phenotypic alterations of the monocyte/myeloid cell compartment in patients with early MS

**Figure 7:**
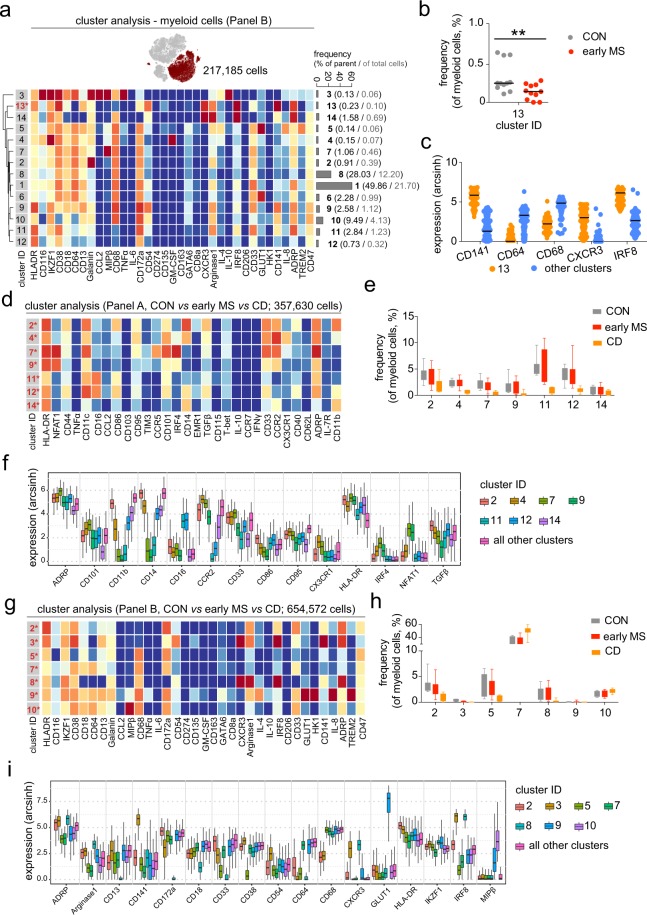
Phenotypic changes in myeloid cell populations from patients with early MS. (**a**) t-SNE map highlights myeloid population (merlot dots). Heat map and cluster analysis of myeloid cells from all samples on the basis of the mean marker expressions (*Panel B*). The bar graph shows mean cluster frequencies as % of parent (black number) and % of total cells (grey number). Cluster 13 (* in red) shows differential abundances between the controls (CON) and patients (early MS). (**b**) The graph shows differences in frequency (%) of cluster 13 between the two studied groups. (**c**) Median expression levels as shown in the dot plot representation for randomly selected cells (n = 256/cluster) in the cluster 13 (orange) and all other clusters (blue). (**d**) Heat map and cluster analysis of differentially abundant subsets of myeloid cells between healthy controls (CON, n = 11), patients with early MS (early MS, n = 11) and Crohn’s disease (CD, n = 8) on the basis of the mean expression of analysed markers using the antibody *Panel A*. (**e**) The graph shows differences in the frequency (%) of all seven differential clusters compared between the three studied groups. (**f**) The Boxplot shows median expression levels of the discriminating markers defining the differentially abundant subsets. The plot was a representation for randomly selected cells (256 cells per cluster) in the seven differential clusters. (**g**) Heat map and cluster analysis of differentially abundant subsets of myeloid cells between the three studied groups on the basis of the mean expression of analysed markers using the antibody *Panel B*. (**h**) The graph shows differences in frequency (%) of all seven differential clusters compared between the three studied groups (CON = grey; early MS = red; CD = orange). (**i**) The Box plot shows median expression levels of the discriminating markers defining the differentially abundant subsets. The plot was a representation for randomly selected cells (256 cells per cluster) in the seven differential clusters. A *P* value < 0.05 was considered statistically significant at 10% FDR, determined using GLMM (**P < 0.01, unadjusted).

Next, we explored the abundance and/or phenotypic changes in the myeloid cell compartment of patients with early MS compared with the control group (Fig. 7). In contrast to previous reports^10–12^, we did not observe major differences in the monocyte/myeloid cell composition between the two groups using antibody *Panel A* (Supplementary Fig. 1). However, results obtained from the profiling using antibody *Panel B* revealed a significant reduction of the frequency of CD141-expressing dendritic cells (DCs, cluster 13) in patients with early MS compared to healthy controls (Fig. 7a–c). This cell subset could be further characterized as CD64^−^CD68^−^CXCR3^+^IRF8^+^ADRP^+^CD38^+^ (Fig. 7a,c). To estimate the degree of phenotypic changes of monocytes in patients with early MS, we compared immune profiles of monocytes from patients with early MS with those from patients with Crohn’s disease (CD) and healthy controls. It is known that monocytes are involved in the immunopathology of inflammatory bowel diseases like CD^19–21^. We detected strong differences in the myeloid cell compartment between patients with CD and early MS or healthy controls using antibody *Panels A* and *B* (Fig. 7d–i). On the basis of the expression levels of markers used in antibody *Panel A*, we identified seven cell subsets in the myeloid compartment that showed differential abundances between the investigated groups (Fig. 7d,e). Furthermore, we defined ADRP, CD101, CD11b, CD14, CD16, CCR2, CD33, CD86, CD95, CX3CR1, HLA-DR, IRF4, NFAT1 and TGFβ1 as markers that were required for the discrimination of these differential cell subsets from the remaining cell subsets (Fig. 7f). Interestingly, all seven differential cell subsets were found to be diminished in the patients with CD (Fig. 7e). Using antibody *Panel B*, we quantified differences in cell frequencies of seven cell subsets (out of 10 defined clusters) in the myeloid compartment of CD patients compared to patients with early MS and healthy controls (Fig. 7g,h). The markers discriminating these differential cell subsets were ADRP, Arginase 1, CD13, CD141, CD172a, CD18, CD33, CD38, CD54, CD64, CD68, CXCR3, GLUT1, HLA-DR, IKFZ1, IRF8 and MIPβ (Fig. 7i). Similar to the significant difference in DCs detected in patients with early MS in comparison to the healthy controls (cluster 13, Fig. 7a), we also found a decreased frequency of CD141^+^CD64^−^CD68^−^CXCR3^+^IRF8^+^ADRP^+^CD38^+^ DCs (cluster 3) in patients with CD. Nevertheless, changes in the immune phenotypes of the myeloid compartment in patients with early MS were minute in comparison to those observed in CD.

## Discussion

MS is an immune-mediated demyelinating and neurodegenerative disease of the CNS with a highly variable disease course. During early MS, immunomodulatory drugs targeting in particular T and B cells are the most effective therapies suggesting strong impacts of these cell populations on the disease. However, it remains unclear whether the aberrant activity of the adaptive arm of the immune system directly contributes to demyelination and/or inflammation of the CNS, or whether a cross-talk between the innate and adaptive arms drives MS pathogenesis^22^. Therefore, comprehensive information about the responses of both adaptive and innate immune cells during early MS will help to better understand the communication between both arms of the immune system, and to potentially manipulate them. In this study, we demonstrate the feasibility of extensive immune profiling by multiplexed single-cell mass cytometry to simultaneously determine phenotypic alterations of innate and adaptive immune cell populations in PBMC samples of patients with early MS compared to healthy controls. We suggest two antibody panels comprising of a total of 64 markers that allow for cell subset identification, as well as determination of phenotypic changes in PBMC samples. Although we did not observe overall differences in PBMC composition and marker expression, high-dimensional cluster analysis revealed changes in the cellular composition of T and B cell subsets, as well as in DCs, suggesting responses of various circulating lymphocyte and myeloid cell subpopulations during early MS.

In the lymphoid cell compartment of patients with early MS, using antibody *Panel A*, we detected increased frequencies of CD4^+^ and CD8^+^ cells that highly expressed CCR7 and were negative for CD95 (Fas). These subsets expressed low levels of NFAT1 and T-bet. In contrast, we observed a decreased abundance of the CCR7^lo^NFAT1^hi^T-bet^hi^ subset of CD4^+^ T cells. These findings underscore the importance of T cell-responses in MS, which is in line with results obtained from EAE model in rodents, which suggested that CCR7 plays a crucial role in T cell homing and neuroinflammation during the priming of autoimmune CD4^+^ T cells but not in the CNS^23^. In fact, specific constitutive deletion of CCR7 on CD4^+^ T cells induced immune tolerance to EAE^23^. T-box transcription factor (T-bet) is expressed in T lymphocytes and committed to T cell development^24^. This transcription factor is recognized as a key regulator for Th1 cell differentiation and is considered to be critical for T cell pathogenicity in EAE since mice deficient in T-bet are resistant to the development of EAE^25^. Also, the nuclear factor of activated T cell (NFAT) transcription factor is involved in the regulation of T-cell development, activation and differentiation^26^. Moreover, our results are in line with observations in patients with relapsing-remitting MS that suggest an increase of circulating CD95^−/lo^CD4^+^ and CD8^+^ T cells compared to healthy controls^27,28^. We also identified increased abundance of IL-6-expressing CD8a^−^CD47^hi^IKZF1^hi^ T cells in the peripheral blood of patients with early MS. This finding is in line with the previous observation of increased CD3^+^CD8^−^ cells producing the Th17-related cytokine IL-6 in inflammatory niches^29^. Moreover, we found reduced frequencies of IRF8^+^HLA-DR^+^ lymphocytes that highly expressing MIPβ. Although B cell markers such as CD19 or CD20 were missing in the antibody *Panel B*, this IRF8^+^HLA-DR^+^MIPβ^+^ cell cluster may be identified as a member of the B cell population. Of note, it has been demonstrated that B cells increased MIPβ (or CCL4) expression upon activation, and that MIPβ plays a crucial role in the recruitment of regulatory T cells. Failure of CCL4-mediated T cell recruitment leads to autoimmune activation^30^. Further, an increased abundance of the B cell subset that was CCR7^+^CD62L^low/−^ was also detected, whereas the proportion of CCR7^+^CD62L^+^ B cells was increased in early MS. Shedding expression of CD62L on B cells, for example after infection, could affect B cell receptor signalling and B cell migration^31^.

In contrast to lymphocytes, massive immune profiling using both antibody panels revealed no phenotypic differences of monocytes in patients with early MS compared to healthy controls. In contrast, patients with Crohn’s disease (CD) showed a strong inflammatory phenotype in monocytes, suggesting that monocyte responses in patients with early MS might be related to the more compartmentalized inflammation in the CNS. Nevertheless, since it has been unequivocally shown in EAE that monocytes critically contribute to the pathophysiology of EAE^32,33^, the contradictory findings reported herein might be explained by a limitation of markers which were analysed in this study. In contrast to single-cell RNA-sequencing (scRNA-Seq) analysis, CyTOF assesses single-cell phenotypes using an antibody-based approach, which is not a completely unbiased method. Further investigations include the extension to novel markers (possibly identified by RNA-sequencing). Notably, we observed decreased abundance of CD141^+^CD68^lo^ myeloid DCs in patients with early MS compared to healthy controls. Interestingly, myeloid DCs have been suggested as critical regulators of regulatory T cell development in MS^34^.

In sum, we performed comprehensive immune profiling of PBMCs at the single-cell level, which allowed us to unambiguously characterise immune phenotypes and functions of diverse cell populations in the peripheral blood of patients with early MS. Our results extend the findings of previous, in particular transcriptomic, studies that suggested multiple cell types, including different subsets of lymphocytes and myeloid DCs, orchestrate the complex immune responses during early MS^5,35–39^ and EAE^40^. These findings underscore the importance of comprehensive immune profiling in diseases with multifaceted aspects of inflammation like MS. The power of high-dimensional single-cell techniques like CyTOF and scRNA-Seq will in the future help to provide a better understanding of the heterogeneity of immune responses in MS.

## Methods

### Subjects

The study was registered and approved by the Ethics Committee of Charité – Universitätsmedizin Berlin. All participants provided written informed consent before any study-related procedures were undertaken. The study has been performed according to the Declaration of Helsinki and to the relevant ethical guidelines for research in humans. Heparinized blood samples were obtained by peripheral venipuncture from 11 patients with early MS and 11 healthy controls, as well as from 8 patients with Crohn’s disease **(**Supplementary Table 1). All patients with early MS included in this work participated in an ongoing prospective observational study of patients with early MS (Berlin CIS Cohort; NCT01371071), which started recruitment in January 2011. Inclusion criteria were: age > 18 years, a first clinical event suggestive of inflammatory demyelination (clinically isolated syndrome (CIS) or early MS) not meeting the McDonald 2010 criteria for relapsing-remitting MS (RRMS)^41^ within six months before inclusion into the study or a diagnosis of RRMS according to the McDonald 2010 criteria within 24 months before inclusion into the study. Exclusion criteria were: a history of alcohol or drug abuse, any conditions precluding magnetic resonance imaging examinations and any ocular diseases precluding optical coherence tomography. Patients investigated in the present analysis were drug naïve at the time of the blood draw. Patients underwent a thorough neurological examination, including determination of the expanded disability status scale (EDSS) score.

### PBMC isolation

PBMCs were isolated from heparinized blood (27 ml) within 4.5 hours of the blood draw through Biocoll (Biochrom GmbH, Berlin, Germany) density centrifugation at 760 × g for 20 minutes at room temperature as described before^42^. The blood mononuclear cell fraction was recovered and washed in phosphate-buffered saline (PBS; Biochrom GmbH) at 560 × g for 20 minutes and at 400 × g for 15 minutes. Cell pellets were stored in liquid nitrogen for further analysis at a concentration of 5 × 10^6^ cells/ml in RPMI-1640 (Gibco) containing 10% dimethyl sulfoxide (Sigma Aldrich Chemie GmbH), 20% fetal bovine serum (Biochrom GmbH) and 1% Hepes (Gibco).

### Live cell barcoding

Individual PBMC samples (~5 × 10^6^ cells) were stained with premade combinations of preconjugated ^89^Y-CD45 (HI30, Fluidigm) and in house-conjugated ^113^In, ^115^In, ^195^Pt, ^196^Pt or ^198^Pt-CD45 (HI30, Biolegend)^13,14^ for 30 minutes at 4 °C. Cells were then washed twice and pooled. The multiplexed samples were then stained for surface and intracellular markers and subsequently measured by mass cytometry.

### Antibodies

Anti-human antibodies (Supplementary Table 2 for *Panel A* **&** Supplementary Table 3 for *Panel B*) were purchased either preconjugated to metal isotopes (Fluidigm) or from commercial suppliers in purified form and conjugated in house using the MaxPar X8 kit (Fluidigm) according to the manufacturer’s protocol. Each antibody was titrated and validated as into the working panels prior to use to ensure that the resulted signals were informative.

### Cell-surface and intracellular staining

After cell barcoding, washing and pelleting, the combined samples were stained and processed as described previously^17^. Briefly, cells were re-suspended in 100 µl of antibody cocktail directed against cell surface markers (Supplementary Tables 2 and 3) and incubated for 30 minutes at 4 °C. Then, cells were washed twice with cell staining buffer (PBS containing 0.5% bovine serum albumin and 2 mM EDTA). For intracellular staining, the stained (non-stimulated) cells were then incubated in fixation/permeabilization buffer (Fix/Perm Buffer, eBioscience) for 60 minutes at 4 °C. Cells were then wash twice with permeabilization buffer (eBioscience). The samples were then stained with antibody cocktails directed against intracellular molecules (Supplementary Tables 2 and 3) in permeabilization buffer for 1 hour at 4 °C. Cells were subsequently washed twice with permeabilization buffer and incubated overnight in 4% methanol-free formaldehyde solution. The fixed cells were then washed and re-suspended in 1 ml iridium intercalator solution (Fluidigm) for 1 hour at room temperature, followed by two washes with cell staining buffer and two washes with ddH_2_O (Fluidigm). Finally, cells were pelleted and kept at 4 °C until CyTOF measurement.

#### Bead staining

For the bead-based compensation of the signal spillover, AbC total antibody compensation beads (Thermo Fisher Scientific) were single stained with each of the antibodies used in *Panel A* and *B* according to manufacturer’s instructions.

#### CyTOF measurement

Cells were analysed using a CyTOF2 upgraded to Helios specifications, with software version 6.7.1014^17^. The instrument was tuned according to the manufacturer’s instructions with tuning solution (Fluidigm) and measurement of EQ four element calibration beads (Fluidigm) containing 140/142Ce, 151/153Eu, 165Ho, and 175/176Lu served as a quality control for sensitivity and recovery.

Directly prior to analysis cells were re-suspended in ddH_2_O, filtered through a 20 µm cell strainer (Celltrix, Sysmex), counted and adjusted to 3–5 × 10^5^ cells/ml. EQ four element calibration beads were added at a final concentration of 1:10 v/v of the sample volume to be able to normalize the data to compensate for signal drift and day-to-day changes in instrument sensitivity.

Samples were acquired with a flow rate of 300–400 events/second. The lower convolution threshold was set to 400, with noise reduction mode turned on and cell definition parameters set at event duration of 10–150 pushes (push = 13 µs). The resulting flow cytometry standard (FCS) files were normalized and randomized using the CyTOF software’s internal FCS-Processing module on the non-randomized (‘original’) data. The default settings in the software were used with time interval normalization (100 seconds/minimum of 50 beads) and passport version 2. Intervals with less than 50 beads per 100 seconds were excluded from the resulting FCS-file.

#### Mass cytometry data processing and analysis

As described previously^17^, Cytobank (www.cytobank.org) was used for initial manual gating on live single cells and boolean gating for de-barcoding. Nucleated single intact cells were manually gated according to DNA intercalators ^191^Ir/^193^Ir signals and event length. For de-barcoding, Boolean gating was used to deconvolute individual sample according to the barcode combination. All de-barcoded samples were then exported as individual FCS files for further analysis. No significant difference in number cell count was detected between the studied groups (cell count per sample (mean ± sd; rang): *Panel A* = 14,246 ± 17,800; 2,800–59,686 (early MS) and 11,174 ± 12,350; 2,370–43,960 (control), and *Panel B* = 25,497 ± 29,518; 2,383–98,367 (early MS) and 20,793 ± 23,592; 3,048–82,960 (control)). Each FCS file was compensated for signal spillover using R package CATALYST^43^ and transformed with arcsinh transformation (scale factor 5) prior to data analysis. Immune phenotypes of PBMCs were visualized using reduced-dimensional (2D) t-SNE maps generated according to the expression level of all markers used in the panel (both *Panel A* and *B*). For embedding, we set hyperparameters to perplexity of 30, theta of 0.5, and iterations of 1000 per 100,000 analysed cells). FCS files containing the t-SNE coordination as additional two parameters were exported from Cytobank for downstream exploratory and statistical analyses using R, as previously described^17,18^. For population identification, *FlowSOM/ConsensusClusterPlus*^18,43,44^ (used all defaults with maxK = 25) clustering was used with 100 initial SOM-grid points and maxim of 25 meta-clusters. At first, we identified clusters of the main PBMC-lineages on the basis of expression levels of HLA-DR, CD19, CD44, CD4, CD11c, CD16, CD3, CD56, CD14, CD8a, T-bet, CD33, CCR2 and CD11b in the *Panel A*; and HLA-DR, CD38, CD64, CD68, CD8a, CD33 and CD64 in the *Panel B*. Subsequently, to obtain high enough resolution for further analysis and calculate frequencies within individual lineages, clusters were manually merged according to consensual marker-expression patterns into subsets representing B cells, CD4/CD8-single positive and double-negative T cells, NK cells, and myeloid cells, respectively. Each individual lineage cluster contained on average at least 200 events. These subsets served as input for second-level *FlowSOM/ConsensusClusterPlus* meta-clustering. Based on visual inspection of t-SNE plots and heat maps generated at each merging step, for each parent subset a final number of meta-clusters was chosen that merged clusters into populations with consistent phenotypes (with a minimal mean frequency of 0.1% of parent). Of note, we followed the concept of over-clustering for second-level meta-clustering, in order to study more specific subpopulations at higher detail in each main subsets (especially in myeloid cells)^18^. The number of defined clusters may not solely represent biologically functional subsets in PBMCs, but it should rather be interpreted as an exploratory tool for discovery of the differential abundance of small cell populations between the two studied groups. Based on visual inspection of t-SNE plots and heat maps generated at each merging step for each parent subset, a final number of meta-clusters was decided to display the merged clusters into populations with consistent phenotypes.

### Statistical analysis

No randomization and blinding strategies were applied in this study. However, data processing and analysis, as well as statistical testing were carried out in an unsupervised manner. Dichotomous variables of the sample cohort were analysed with Fisher’s exact test (GraphPad Prism). Quantitative data are shown as independent data points with median or Box-Whisker-Plot. Analyses of statistical significance were performed by computational analysis using generalized linear mixed-effects model (GLMM) available through R package *diffcyt* (used all defaults with analysis_type = “DA”, method_DA = “diffcyt-DA-GLMM”, min-cells = 3) and false discovery rate (FDR) adjustment (at 10% using Benjamini-Hochberg procedure) for multiple hypothesis testing. A *P* value < 0.05 (unadjusted) at 10% FDR was considered statistically significant. In addition, a cluster was defined as differentially abundant if it presented in at least 80% of the samples (of each or either (in the case of a disease/control-specific cluster) studied group).

### Ethics approval and consent to participate

The study was registered and approved by the Ethics Committee of Charité – Universitätsmedizin Berlin, Berlin, Germany.

## Supplementary information

Supplementary Information

## Data Availability

The datasets used and/or analysed during the current study are available from the corresponding author on reasonable request that does not include confidential patient information.
