# Supplementary material for: Multi-parameter immune profiling of peripheral blood mononuclear cells by multiplexed single-cell mass cytometry in patients with early multiple sclerosis
Source: Sci Rep. 2019 Dec 19;9:19471. doi: 10.1038/s41598-019-55852-x (PMC6923404; doi:10.1038/s41598-019-55852-x)
Supplement: Supplementary file 1 — Supplementary Information [file 41598_2019_55852_MOESM1_ESM.pdf]

Supplementary information

Multi-parameter immune profiling of peripheral blood mononuclear cells by multiplexed single-cell mass cytometry in patients with early multiple sclerosis

Chotima Böttcher[1†*], Camila Fernández-Zapata[1†], Stephan Schlickeiser[2†], Desiree Kunkel[2], Axel R Schulz[3], Henrik E Mei[3], Carl Weidinger[4], René M. Gieß[5,6], Susanna Asseyer[5], Britta Siegmund[4], Friedemann Paul[5,6,7,8], Klemens Ruprecht[6†], Josef Priller[1,8,9,10†*]

[1]Department of Neuropsychiatry and Laboratory of Molecular Psychiatry, [2]Berlin-Brandenburg Center for Regenerative Therapies (BCRT), Charité – Universitätsmedizin Berlin, Berlin, Germany. [3]German Rheumatism Research Center (DRFZ), Berlin, Germany. [4]Medical Department for Gastroenterology, Division of Gastroenterology, Infectiology and Rheumatology, Charité – Universitätsmedizin Berlin, Berlin, Germany. [5]NeuroCure Clinical Research Center (NCRC), [6]Department of Neurology and Clinical and Experimental Multiple Sclerosis Research Center, Charité – Universitätsmedizin Berlin, corporate member of Freie Universität Berlin, Humboldt-Universität zu Berlin, and Berlin Institute of Health, Berlin, Germany. [7]Experimental and Clinical Research Center, Max Delbrueck Center for Molecular Medicine and Charité – Universitätsmedizin Berlin, Berlin, Germany. [8] Berlin Institute of Health, [9]DZNE, Berlin, Germany. [10]University of Edinburgh and UK Dementia Research Institute, Edinburgh, UK.

Supplementary Table 1 Demographic information

group	*N*	f/m	Age (years, mean (range))	EDSS (mean (range))
CON	11	10/1	36 (24-56)	n.a.
early MS	11	8/3	36 (26-51)	0.9 (0-2)
CD	8	5/3	38 (27-49)	n.a.
p value				-
early MS *vs* CON	-	$p = 0.5865$	$p = 0.9529$	-
CD *vs* CON	-	$p = 0.2621$	$p = 0.6840$	-
early MS *vs* CD	-	$p = 1.0000$	$p = 0.5618$	-

CD: Crohn's disease; CON: Healthy controls; MS: Multiple Sclerosis; CIS = Clinically Isolated Syndrome; EDSS: Expanded disability status scale

Supplementary Table 2 The list of antibodies and barcoding reagents – *Panel A*

isotope tag	target	clone / company
^{89}Y	CD45 (live barcode)	HI30 / Fluidigm
^{113}In	CD45 (live barcode)	HI30 / Biolegend
^{115}In	CD45 (live barcode)	HI30 / Biolegend
^{141}Pr	HLA-DR	L243 / Fluidigm
^{142}Nd	CD19	HIB19 / Fluidigm
^{143}Nd	NFAT1	D43B1 / Fluidigm
^{144}Nd	CD44	BJ18 / Biolegend
^{145}Nd	CD4	RPA-T4 / Biolegend
^{146}Nd	TNFα	Mab11 / Fluidigm
^{147}Sm	CD11c	Bu15 / Fluidigm
^{148}Nd	CD16	3G8 / Fluidigm
^{149}Sm	CCl2	5D3-F7 / Biolegend
^{150}Nd	CD86	IT2.2 / Biolegend
^{151}Eu	CD103	Ber-ACT8 / Fluidigm
^{152}Sm	CD95	DX2 / Biolegend
^{153}Eu	TIM3	F38-2E2 / Biolegend
^{154}Sm	CD3	UCHT1 / Fluidigm
^{155}Gd	CD56	B159 / Fluidigm
^{156}Gd	CCR5	NP-6G4 / Fluidigm
^{158}Gd	CD101	BB27 / Fluidigm
^{159}Tb	IRF4	3E4 / Biolegend
^{160}Gd	CD14	RMO52 / eBioscience
^{161}Dy	EMR1 (F4/80)	A10 / Bio-Rad
^{162}Dy	CD8a	RPA-T8 / Fluidigm
^{163}Dy	TGF-β	TW4-2F8 / Biolegend
^{164}Dy	CD115	9-4D2-1E4 / Biolegend
^{165}Ho	T-bet (PE)	eBio4B10/ eBioscience
^{166}Er	IL-10	JES3-9D7 / Fluidigm
^{167}Er	CCR7	G043H7 / Fluidigm
^{168}Er	IFNγ	B27 / Fluidigm
^{169}Tm	CD33	WM53 / Fluidigm
^{170}Er	-	-
^{171}Yb	CCR2	K036C2 / Biolegend
^{172}Yb	CX3CR1	2A9-1 / Fluidigm
^{173}Yb	CD40	5C3 / Biolegend
^{174}Yb	CD62L	DREG-56 / Biolegend
^{175}Lu	ADRP	polyclonal / non commercial
^{176}Yb	IL-7R	A019D5 / Fluidigm
^{195}Pt	CD45 (live barcode)	HI30 / Biolegend
^{196}Pt	CD45 (live barcode)	HI30 / Biolegend
^{198}Pt	CD45 (live barcode)	HI30 / Biolegend
^{191}Ir	DNA	- / Fluidigm
^{193}Ir	DNA	- / Fluidigm
^{209}Bi	CD11b	ICRF44 / Fluidigm

Supplementary Table 3 The list of antibodies and barcoding reagents – *Panel B*

isotope tag	target	clone / company
^{89}Y	CD45 (live barcode)	HI30 / Fluidigm
^{113}In	CD45 (live barcode)	HI30 / Biolegend
^{115}In	CD45 (live barcode)	HI30 / Biolegend
^{141}Pr	HLA-DR	L243 / Fluidigm
^{142}Nd	CD116	4HI / Biolegend
^{143}Nd	IKZF1	Ikaros / Fluidigm
^{144}Nd	CD38	HIT2 / Fluidigm
^{145}Nd	CD18	TS1/18 / Fluidigm
^{146}Nd	CD64	10.1 / Fluidigm
^{147}Sm	CD13	WM15 / FLuidigm
^{148}Nd	Galanin	581403 / Fluidigm
^{149}Sm	CCl2	5D3-F7 / Biolegend
^{150}Nd	MIPβ	D21-1351 / Biolegend
^{151}Eu	CD68	Y1/82A / Fluidigm
^{152}Sm	TNFα	Mab11 / Fluidigm
^{153}Eu	IL-6	MQ2-13A5 / Biolegend
^{154}Sm	CD172a	15-414 / Biolegend
^{155}Gd	CD54 (ICAM1)	HA58 / Biolegend
^{156}Gd	PD-L1	29E.2A3 / Fluidigm
^{158}Gd	CD135	BB27 / Fluidigm
^{159}Tb	GM-CSF	BVD2-21C11 / Biolegend
^{160}Gd	CD163	GHI/61 / Biolegend
^{161}Dy	GATA6	D61E4 / Fluidigm
^{162}Dy	CD8a	RPA-T8 / Fluidigm
^{163}Dy	CXCR3	G025H7 / Fluidigm
^{164}Dy	Arginase-1	658922 / Fluidigm
^{165}Ho	IL-4 (PE)	8D4-8 / Biolegend
^{166}Er	IL-10	JES3-9D7 / Fluidigm
^{167}Er	IRF8	7G11A45 / Biolegend
^{168}Er	CD206	15-2 / Fluidigm
^{169}Tm	CD33	WM53 / Fluidigm
^{170}Er	-	-
^{171}Yb	GLUT1	195205 / R&D Systems
^{172}Yb	HK1	3A10 / Thermo Fisher Scientific
^{173}Yb	CD141	1A4 / Biolegend
^{174}Yb	IL-8 (FITC)	EH12.2H7 / Fluidigm
^{175}Lu	ADRP	polyclonal / non commercial
^{176}Yb	TREM2	237920 / R&D Systems
^{195}Pt	CD45 (live barcode)	HI30 / Biolegend
^{196}Pt	CD45 (live barcode)	HI30 / Biolegend
^{198}Pt	CD45 (live barcode)	HI30 / Biolegend
^{191}Ir	DNA	- / Fluidigm
^{193}Ir	DNA	- / Fluidigm
^{209}Bi	CD47	CC2C6 / Fluidigm

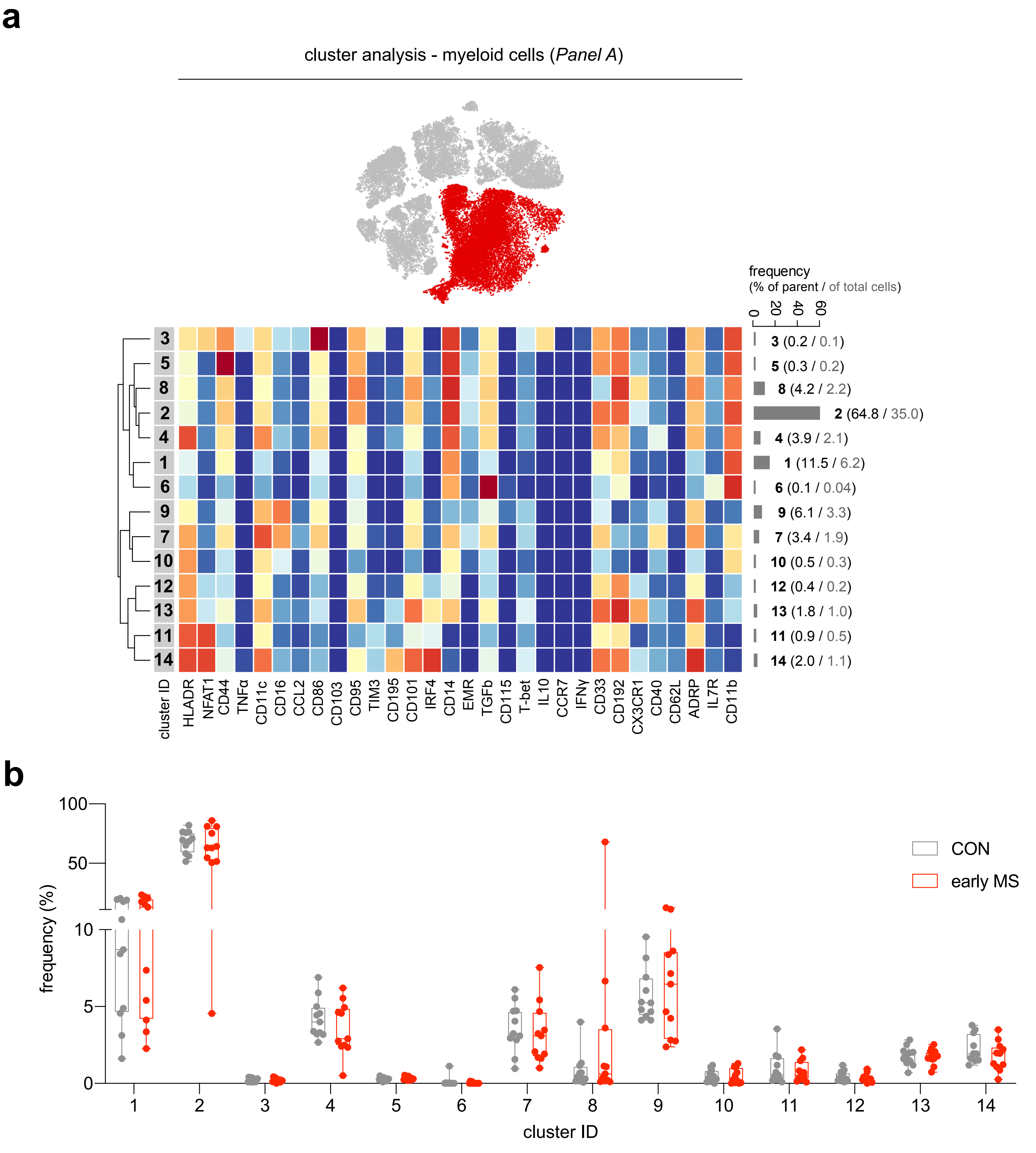

Supplementary Figure 1 Meta-cluster abundances of the myeloid cell subset (*Panel A*). (**a**) Heat map and cluster analysis of the myeloid cell population from all samples on the basis of the mean expression of analysed markers using antibody *Panel A*. Identified clusters are indicated by dendrograms. Heat colours show overall marker expression levels (red, high expression; dark blue, no expression). The bar graph shows mean frequency (all samples) of each identified cluster as a % of parent (number in black) and a % of total cells (number in gray). (**b**) The Boxplot shows distribution of cluster frequencies (of parent) of the 14 identified clusters of each individual sample in both studied groups (CON, gray; early MS, red). No significant differences in the cluster distribution was observed between the studied group.
